# The Human Microbiome, an Emerging Key-Player in the Sex Gap in Respiratory Diseases

**DOI:** 10.3389/fmed.2021.600879

**Published:** 2021-05-07

**Authors:** Clémence Beauruelle, Charles-Antoine Guilloux, Claudie Lamoureux, Geneviève Héry-Arnaud

**Affiliations:** ^1^Univ Brest, Inserm, EFS, UMR 1078, GGB, Brest, France; ^2^Unité de Bactériologie, Pôle de Biologie-Pathologie, Centre Hospitalier Régional et Universitaire de Brest, Hôpital de la Cavale Blanche, Brest, France

**Keywords:** microbiome, lung, gut, respiratory diseases, sex gap

## Abstract

The sex gap is well-documented in respiratory diseases such as cystic fibrosis and chronic obstructive pulmonary disease. While the differences between males and females in prevalence, severity and prognosis are well-established, the pathophysiology of the sex difference has been poorly characterized to date. Over the past 10 years, metagenomics-based studies have revealed the presence of a resident microbiome in the respiratory tract and its central role in respiratory disease. The lung microbiome is associated with host immune response and health outcomes in both animal models and patient cohorts. The study of the lung microbiome is therefore an interesting new avenue to explore in order to understand the sex gap observed in respiratory diseases. Another important parameter to consider is the gut-lung axis, since the gut microbiome plays a crucial role in distant immune modulation in respiratory diseases, and an intestinal “microgenderome” has been reported: i.e., sexual dimorphism in the gut microbiome. The microgenderome provides new pathophysiological clues, as it defines the interactions between microbiome, sex hormones, immunity and disease susceptibility. As research on the microbiome is increasing in volume and scope, the objective of this review was to describe the state-of-the-art on the sex gap in respiratory medicine (acute pulmonary infection and chronic lung disease) in the light of the microbiome, including evidence of local (lung) or distant (gut) contributions to the pathophysiology of these diseases.

## Introduction

Sexual dimorphism is a characteristic feature of many major diseases, including in asthma, chronic obstructive pulmonary disease (COPD), cystic fibrosis (CF), and non-CF-related bronchiectasis. In these chronic respiratory diseases, female patient generally have more severe symptoms, poorer quality of life, and greater mortality (1–3). On the opposite, acute pneumonia are associated to worse prognoses in male patient than in female (2). While sex differences in prevalence, severity, and prognosis of these diseases are well-documented, the pathophysiology of the sex difference has been poorly characterized to date.

In recent years, next-generation sequencing (NGS) technologies have changed the way we view the microbial communities that inhabit every mucosal surface of the human body, including compartments previously considered sterile such as the lung. These microbiomes are increasingly studied as they provide many keys to understanding organ homeostasis and disease pathophysiology. Most research has focused on the gut microbiome and its relation to disease (4). More recently, it has been shown that the airways, like the gut, harbor a unique steady-state microbiome, and that dysbiosis in the lung microbiome influences respiratory health and disease (5).

Sexual dimorphism is found in many diseases, and a microbial sex gap, also called “microgenderome,” was described at several body sites [gut, oral (palatine tonsils, tongue, saliva), skin (retro-auricular crease and antecubital fossa), and upper respiratory airways sites (anterior nares)], with potential health impact (6–11). The objective of this review is to provide an update of sex gap in respiratory diseases. We investigate the potential microbiome contribution [including evidence of local (lung) and distant (gut) microbiome] to the physiopathology of respiratory diseases, for which female sex is in most cases, a risk factor.

## Women and Men are Not Equal in the Face of Respiratory Diseases

Differences between men and women have been highlighted in numerous respiratory diseases. Being a woman is either an advantage or a disadvantage, depending on the disease.

Women are more at risk of chronic pulmonary diseases, except for lung cancer, where men tend to show greater mortality and incidence (12). In adults, asthma is more frequent in women, who present more respiratory symptoms and greater morbidity (2, 13–15). Interestingly, during childhood, asthma is more frequent in boys than in girls (16); then the pattern reverses around 16–18 years of age (17). In adulthood, in addition to a higher prevalence, asthma is more severe in women, who tend to have higher healthcare use, poor asthma control, and a higher number of severe exacerbations leading to hospitalization than men (18–20). In adulthood, a sex-based difference is also observed in COPD with regard to clinico-radiographic phenotype, symptom severity, and quality of life. Female patients are more sensitive to the adverse effects of smoking, have more symptoms such as dyspnea or cough, and have a higher proportion of bronchiectasis (3, 21, 22).

In non-CF-related bronchiectasis, prevalence is higher in men but severity is greater in women (23). In CF, being a woman incurs greater risk of mortality (1). CF women had lower median life expectancy than men (36.0 vs. 38.7 years) (1) and female sex was shown to be a significant risk factor for death (1). Women also acquire *Pseudomonas aeruginosa* (PA) at an earlier age, as well as several other pathogens such as *Staphylococcus aureus, Haemophilus influenzae, Achromobacter xylosoxydans, Burkholderia cepacia, Aspergillus* and non-tuberculous mycobacteria (NTM) (1). CF women also have a higher risk of non-mucoid to mucoid conversion of PA (1). This sex effect in CF was confirmed in mouse models, where females inoculated with PA died earlier and showed slower bacterial clearance than males (24). A sex gap was observed in CF patients' response to ivacaftor, a CFTR (CF transmembrane conductance regulator) modulator: although there was no significant sex difference in FEV-1 improvement, ivacaftor-treated women showed greater reduction in pulmonary exacerbation and in sweat chloride than men (25).

In respiratory tract infection, it is male sex that is a risk factor for acute infection (26). The incidence of community-acquired pneumonia, including *Streptococcus pneumoniae, Chlamydophila pneumoniae*, and *Legionella pneumophila* infection, is higher in men than in women. Epidemiological findings on the COVID-19 pandemic in various parts of the world indicated higher morbidity and mortality in males than females (27). Within mycobacterial infections, there is a dichotomy between *Mycobacterium tuberculosis* and NTM, tuberculosis affecting more men than women, with higher risk of mortality (26), while NTM infection is more common in slender, older women without any overt immune defects (28).

## Sex-Oriented Microbiome in Pulmonary Disease: a Two-Site Story

**Figure 1:**
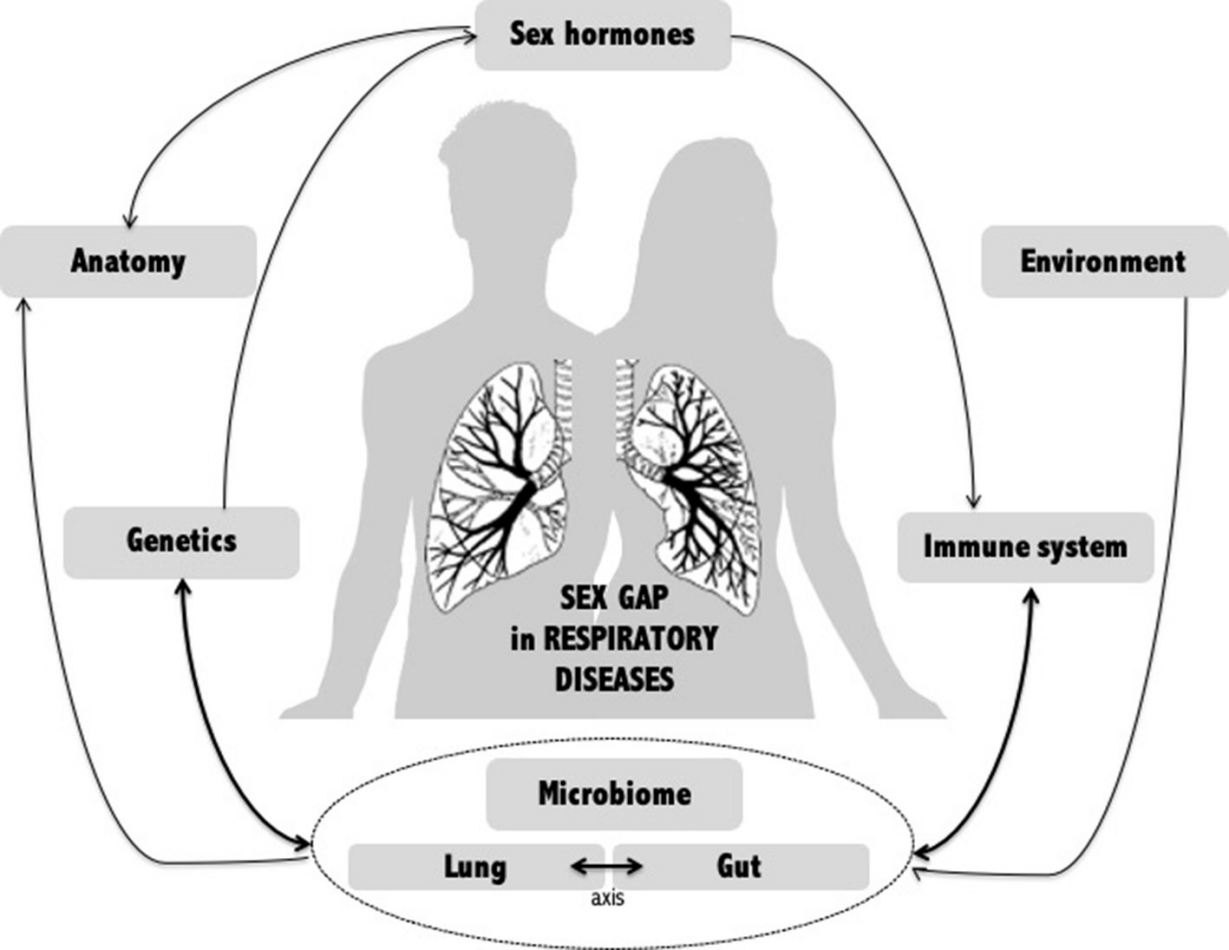
Potential mechanisms of gender gap in respiratory diseases. Multiple factors have been examined to explain the sex gap in respiratory diseases. Among them, the human microbiome, whether local (lung) or distal (intestine), appears to be an emerging key player linking many other factors.

A wide range of factors have been examined to explain the sex gap in airway diseases (Figure 1). To date, no single explanation accounts for this sex difference, which appears to be multifactorial. However, recent data suggest that the human microbiome may be a key player. The lung microbiome is, of course, essential in lung issues, but emerging data on the gut-lung axis show that it is also important to take stock of the involvement of the gut microbiome in the sex gap observed in lung disease.

### Microgenderome of the Lung Niche

As dysbiosis in the airway microbiome influences respiratory health and disease (5), sex differences in the airway microbiome likely contribute to differential expression of respiratory diseases according to sex. Many lung microbiome-based cohort studies included both male and female patients, but few investigated or even discussed a potential sex gap. Sex was considered more as a standardizing variable than as a focus in itself. Moreover, animal studies are generally carried out only in animals of the same sex.

Only one study of lower respiratory tract infection clearly concluded that sex did not correlate with microbiome composition (29). This study was conducted in a pediatric cohort, which may explain the lack of difference between the sexes. The lung microbiome has been shown to evolve with age (30), and sex-oriented morphological modifications occur in the lung during life (11, 31).

In mouse models of lung inflammation, Barfod et al. used denaturating gradient gel electrophoresis to investigate lung microbiome changes associated with inflammation (32), and observed different lung microbiome profiles according to sex. All mice grew up in the same boxes, inhaling the same air and eating the same food; molecular analysis was performed with the same kits, and procedures were standardized to limit bias in microbiome description. The study showed that the lung microbiome is sex-dependent, and not just a reflection of the gut microbiome.

In mice fed with vitamin-D-enriched diet throughout life, a sex difference in the lung microbial community was observed, with higher rates of *Acinetobacter* in female than male lungs (33). No sex effect was observed in chronically vitamin-D-deficient mice. Likewise, no effect of vitamin D on the lung microbiome was observed in initially deficient mice with secondary supplementation. *Acinetobacter* may colonize the lungs early in life and has been linked to allergy protection (33). These data are consistent with the window of opportunity to intervene on the lung microbiome being in the early weeks of life (34).

NGS approaches have allowed further exploration of the sex specificities of the microbiome in humans. Data from the Human Microbiome Project (HMP) were reanalyzed to decipher sex differences (11). Although the lower airway microbiome was not studied in the HMP, the upper airway (anterior nares) microbiome differed between the sexes. Comparisons of community diversity revealed greater diversity in the upper airways of men with three types of analysis: (1) The whole community. (2) The five main phyla (higher diversity for men for the phylum Actinobacteria). (3) The core and accessory microbiomes. This gender difference was also observed at the species composition level. Focusing on species interaction, the central nodes (species) and skeleton (species backbone) can be selected by sex, including in the upper respiratory tract, resulting in a different core/periphery species composition between the two sexes.

In a study investigating the impact of inhaled aztreonam on the lung microbiome in CF, sex analysis revealed differences in alpha diversity, and gender-specific OTUs and genera (35). Males had a more diverse microbiome (significant higher Shannon diversity index), correlating with a proportionally lower abundance of PA and increased abundance in other genera, including *Streptococcus, Dialister, Shuttleworthia*, and *Stenotrophomonas*. In contrast, females showed a higher abundance of *Pseudomonas* and a tendency toward greater responsiveness to aztreonam. However, the authors did not rule out the possibility that these differences were due to inter-patient variability.

A study compared the lung microbiome in cohorts of patients suffering from lung adenocarcinoma (LUAD) and lung squamous cell carcinoma (LUSC) (12). It was found that the tumor contained unique, significantly deregulated microbes according to type of lung cancer and to sex. In young LUSC male patients, the most prominent bacterial species was *Pseudomonas putida*. Likewise, all LUAD females exhibited uniquely implicated bacterial species, different from those of LUAD males (12). These uniquely implicated bacterial species may constitute accurate diagnostic biomarkers for lung cancer type, as they are adapted to the patient's sex.

### Microgenderome of the Gut Niche

Another important parameter to consider is the gut–lung axis. The role of the gut microbiome in local health homeostasis and disease is now widely recognized. But, apart from its local impact, the gut microbiome also influences distant organs, including lungs (36). Accumulating evidence highlighted the influence of the gut microbiome on lung immunity, giving rise to the concept of the gut–lung axis (36, 37). This gut-lung axis consists in vital bidirectional cross-talk, allowing passage of microbial metabolites, including short-chain fatty acids, reported to be key local and systemic signaling molecules sustaining immune and tissue homeostasis (38–40). Thus, disturbance in this bidirectional exchange is linked to increased emergence of airway diseases, including asthma, COPD, CF, lung cancer, or respiratory infection (36, 37, 41). In the light of these data, the hypothesis is that sexual dimorphism in the gut microbiome may contribute to the sex gap observed in respiratory diseases.

Based on the Human Microbiome Project (HMP) data, Ding and Schloss highlighted a sex gap in 300 healthy adults (9): men were three times more likely than women to harbor stool community type D, characterized by fewer *Bacteroides* and higher levels of *Prevotella* (9). More recently, a new analysis of the HMP data assessed sex differences in community diversity and composition, confirming a sex gap in various organs, including the gut (11). The lung microbiome was not included in the HMP project, which is why microbiome data are much fewer for this niche.

Although the gut microbiome is the most widely tracked, a sex gap was not considered in most cohort studies: as mentioned above, sex tends to be seen as an adjustment variable rather than an endpoint, and it is murine models that provide most of the data on the sex gap in the gut microbiome.

The first suggestion of a sex bias in the commensal microbiome concerned a mouse model of autoimmune disease (42). In non-obese diabetic (NOD) mice that spontaneously developed type 1 diabetes (T1D), the incidence of T1D was almost double in females compared with males. In contrast, germ-free mice lost this sex bias and male-to-female microbiome transfer led to testosterone-dependent attenuation of autoimmune phenotypes and protection against T1D (42). In this specific model, metagenomic analysis revealed that the microbiome was sex biased, and that the male microbiome became less diverse than the female at puberty (8).

Concerning the link between the respiratory tract and the gut microbiome, a sex difference was reported in human response to ozone (43, 44). Ozone is a common air pollutant, which causes airway hyper-responsiveness, a defining feature of asthma. In a mouse model, a sex gap in the gut microbiome was reported in animals tested for pulmonary response to ozone (45–48), and was thought to contribute to the sex difference in pulmonary response to ozone (45). Male mice developed greater hyper-reactivity to ozone, but this difference was abolished after antibiotic ablation of the gut microbiome (46). Interestingly, ozone response was increased in females housed in cages conditioned by males: i.e., in females exposed to male feces, as mice are coprophagic. The differences in gut microbiome consisted in greater abundance of the *Christensenellaceae* family but smaller abundance of the *Streptococcaceae* family and *Bacteroides acidifaciens* species (46).

## Microbiome, Hormones, and Immune System: a Balanced Triangle

A wide range of factors have been examined to explain the sex differences found in respiratory disease, including sex steroid hormones such as progesterone, estradiol and testosterone (2). These play a number of physiological roles, including modulation of the immune response implicated in chronic airway disease and pulmonary infection (49). Sex steroid hormones regulate the activity of immune cells, including lymphocytes, macrophages, granulocytes, and mast cells (49). Globally, testosterone decreases innate immune system activation in response to pathogen challenge, whereas estrogens enhance cell-mediated and humoral immune response. The differential susceptibility of males to acute bacterial infections may be due to their usually lower immune response.

In addition to acting on immunity, estrogen also has a role in mucus. Mucus plays a central role in protecting the airways from bacterial infection. The main components of mucus are mucins, and in particular the two secreted mucins MUC5AC and MUC5B (50). MUC5AC and MUC5B participate in mucociliary transport and facilitate airway clearance. However, mucus hyper-secretion is deleterious in multiple chronic respiratory diseases such as CF or COPD (51). Both MUC5AC and MUC5B are upregulated by estradiol (21, 50). Thus, estradiol, by causing excessive mucus production, could be a detrimental factor in chronic lung infections. Furthermore, by increasing the hydrophobic properties of these mucins (by increasing the total fucose residues), estradiol inhibits mucociliary clearance, which may also have a negative impact on women with chronic lung disease. With regard to the gut niche, mucins also have an important impact on the composition of the microbiome (52). In the lung as in the gut, mucins represent a continuous source of nutrients for bacteria. Fermentation of mucins generates short-chain fatty acids (SCFAs) and amino acids that are used by mucin-degrading species themselves, but also by opportunistic lung pathogens like PA (53). The concentration of SCFAs has been shown to have a direct impact on bacterial growth (54). High concentrations of SCFAs would impair PA growth, while low concentrations would allow a transient increase in PA growth. In total, the effect of estradiol is potentially harmful to women. It is involved in the hyperproduction of inadequate mucus which could, in addition, represent a potential source of nutrients for pathogenic bacteria. Sex steroid hormones are also involved in communication between microbial pathogens and host (49). This has been shown in CF, where women are more at risk of PA infection. In a retrospective cohort, Chotirmall et al. found that non-mucoid PA predominated in sputum during luteal phase exacerbations (characterized by low estradiol rates) and that more mucoid PA strains were isolated during exacerbations in the follicular phase (high estradiol rates) (55). These findings were consistent with the changes in lung function over the menstrual cycle in CF women (56), with significantly higher FEV-1 in the luteal phase compared to ovulation and menstruation phases. Inflammatory biomarkers and lung function are thus associated with hormonal cycling. In a prospective study, Holtrop et al. followed 23 women with CF who were not taking hormonal contraceptives (57). At the time of estrogen peak (ovulation), there was a significant increase in pro-inflammatory cytokines in sputum (neutrophil elastase) and a decrease in lung function. The introduction of a standard oral contraceptive pill combining estrogen and progesterone was associated with an improvement in pro-inflammatory cytokines (IL-8, TNF-α, neutrophil elastase). Recently, Shaffer et al. described an association between estrogen therapy and first PA isolation in an 18-year-old affirmed woman (transgender) despite improvement in lung function with the CFTR modulator (58). Estrogen level seems to act on survival rates in CF by modifying PA physiology. Estradiol and estriol induced mucoid conversion of PA in CF women, through the mutation of *muc*A gene in the PA genome, as demonstrated *in vitro* (55). This molecular mechanism was associated with selectivity for mucoid isolation, increased exacerbations, and mucoid conversion *in vivo* (55). These results are consistent with other *in vitro* studies that demonstrated that estrogen increased secretion of PA virulence factors such as pyocyanin, and increased PA motility, biofilm formation, swarming, twitching motility, adherence, and invasion of bronchial epithelial cells (59). Experiments in a murine PA infection model showed that ovariectomized mice supplemented with 17β-estradiol succumbed to PA challenge earlier than progesterone or vehicle supplemented mice (24). In male mice, 17β-estradiol supplementation increased the severity of the infection. Two potential mechanisms were suggested: enhancement of Th-17-regulated inflammation, and suppression of innate antibacterial defense (60). Neutrophils treated with 17β-estradiol exhibited an enhanced oxidative burst but decreased PA killing ability and earlier cell necrosis. This hypothesis is borne out by the fact that the estrogen receptor antagonist ICI 182,780 improved survival in female mice infected with PA and restored neutrophil function (24).

This hormonal clue could lead to a means of narrowing the sex gap in pulmonary disease. However, sex steroid hormones as a link between risk of microbial infection and sex are an incomplete explanation as, in several respiratory diseases such as CF, the sex gap is observed before puberty (61) or after menopause (55).

## Conclusion

The sex gap is now well-described in respiratory diseases, with male sex as a pejorative factor for acute lung infection, and female sex for chronic respiratory diseases. The pathophysiology of the sex gap in respiratory diseases, however, remains largely unexplained. Interestingly, as microbial communities came to be increasingly described in various body sites, a sex gap was also discovered in human microbiomes, especially in the gut niche. However, the “microgenderome” associated with respiratory diseases is still poorly characterized. The role of the lung microbiome, which has a strikingly different microbial community and functional repertoire from the gut microbiome, remains to be explored from a sex point of view. Future investigations of the microbiome in respiratory diseases need to stratify data for sex differences in order to better decipher the intricate relationships between microbiome, sex steroid hormones and immune system.

Prioritizing the role of sex in pathophysiological processes is crucial for effective prevention, diagnosis, prognosis and treatment. In this regard, the microbiome-based approach has become a powerful tool to identify sex-specific disease markers. In the near future, it will be crucial to understand local (lung) and distant (gut) microbial contributions to the pathophysiology of respiratory disease (62). This should enable the role of sex-associated microbial taxa to be deciphered as powerful prognostic biomarkers and/or potential health promoters in chronic respiratory disease. It should also enable personalized health-care that takes account of the specificities of women's pathophysiology.

## Author Contributions

GH-A conceived the idea of the article and its plan. CB, C-AG, and GH-A wrote the article. CB made the figure. C-AG and CL contributed to the bibliography search and the final manuscript. All authors contributed to the article and approved the submitted version.

## Conflict of Interest

The authors declare that the research was conducted in the absence of any commercial or financial relationships that could be construed as a potential conflict of interest.

## References

[1] Harness-Brumley CL Elliott AC Rosenbluth DB Raghavan D Jain R. Gender differences in outcomes of patients with cystic fibrosis. J Womens Health. (2014) 23:1012–20. 10.1089/jwh.2014.4985 25495366 PMC4442553

[2] Raghavan D Jain R. Increasing awareness of sex differences in airway diseases. Respirology. (2016) 21:449–59. 10.1111/resp.12702 26677803

[3] Gut-Gobert C Cavaillès A Dixmier A Guillot S Jouneau S Leroyer C. Women and COPD: do we need more evidence? Eur Respir Rev. (2019) 28:180055. 10.1183/16000617.0055-2018 30814138 PMC9488562

[4] Marchesi JR Adams DH Fava F Hermes GDA Hirschfield GM Hold G. The gut microbiota and host health: a new clinical frontier. Gut. (2016) 65:330–9. 10.1136/gutjnl-2015-309990 26338727 PMC4752653

[5] Marsland BJ Gollwitzer ES. Host-microorganism interactions in lung diseases. Nat Rev Immunol. (2014) 14:827–35. 10.1038/nri3769 25421702

[6] Mueller S Saunier K Hanisch C Norin E Alm L Midtvedt T. Differences in fecal microbiota in different European study populations in relation to age, gender, and country: a cross-sectional study. Appl Environ Microbiol. (2006) 72:1027–33. 10.1128/AEM.72.2.1027-1033.2006 16461645 PMC1392899

[7] Flak MB Neves JF Blumberg RS. Immunology. Welcome to the microgenderome. Science. (2013) 339:1044–5. 10.1126/science.1236226 23449586 PMC4005781

[8] Yurkovetskiy L Burrows M Khan AA Graham L Volchkov P Becker L. Gender bias in autoimmunity is influenced by microbiota. Immunity. (2013) 39:400–12. 10.1016/j.immuni.2013.08.013 23973225 PMC3822899

[9] Ding T Schloss PD. Dynamics and associations of microbial community types across the human body. Nature. (2014) 509:357–60. 10.1038/nature13178 24739969 PMC4139711

[10] Dominianni C Sinha R Goedert JJ Pei Z Yang L Hayes RB. Sex, body mass index, and dietary fiber intake influence the human gut microbiome. PLoS ONE. (2015) 10:e0124599. 10.1371/journal.pone.0124599 25874569 PMC4398427

[11] Ma ZS Li W. How and why men and women differ in their microbiomes: medical ecology and network analyses of the microgenderome. Adv Sci. (2019) 6:1902054. 10.1002/advs.201902054 31832327 PMC6891928

[12] Wong LM Shende N Li WT Castaneda G Apostol L Chang EY. Comparative analysis of age- and gender-associated microbiome in lung adenocarcinoma and lung squamous cell carcinoma. Cancers. (2020) 12:1447. 10.3390/cancers12061447 32498338 PMC7352186

[13] PausJenssen ES Cockcroft DW. Sex differences in asthma, atopy, and airway hyperresponsiveness in a university population. Ann Allergy Asthma Immunol. (2003) 91:34–7. 10.1016/S1081-1206(10)62055-8 12877446

[14] Tam A Morrish D Wadsworth S Dorscheid D Man SFP Sin DD. The role of female hormones on lung function in chronic lung diseases. BMC Womens Health. (2011) 11:24. 10.1186/1472-6874-11-24 21639909 PMC3129308

[15] Leynaert B Sunyer J Garcia-Esteban R Svanes C Jarvis D Cerveri I. Gender differences in prevalence, diagnosis and incidence of allergic and non-allergic asthma: a population-based cohort. Thorax. (2012) 67:625–31. 10.1136/thoraxjnl-2011-201249 22334535

[16] Genuneit J. Sex-specific development of asthma differs between farm and nonfarm children: a cohort study. Am J Respir Crit Care Med. (2014) 190:588–90. 10.1164/rccm.201403-0428LE 25171311

[17] Debley JS Redding GJ Critchlow CW. Impact of adolescence and gender on asthma hospitalization: a population-based birth cohort study. Pediatr Pulmonol. (2004) 38:443–50. 10.1002/ppul.20108 15690559

[18] Schatz M Camargo CA. The relationship of sex to asthma prevalence, health care utilization, and medications in a large managed care organization. Ann Allergy Asthma Immunol. (2003) 91:553–8. 10.1016/S1081-1206(10)61533-5 14700439

[19] Zein JG Udeh BL Teague WG Koroukian SM Schlitz NK Bleecker ER. Impact of age and sex on outcomes and hospital cost of acute asthma in the United States, 2011-2012. PLoS ONE. (2016) 11:e0157301. 10.1371/journal.pone.0157301 27294365 PMC4905648

[20] Senna G Latorre M Bugiani M Caminati M Heffler E Morrone D. Sex Differences in severe asthma: results from severe asthma network in Italy-SAN. Allergy Asthma Immunol Res. (2021) 13:219. 10.4168/aair.2021.13.2.219 33474857 PMC7840868

[21] Choi JY Kim SY Lee JH Park YB Kim YH Um SJ. Clinical characteristics of chronic obstructive pulmonary disease in female patients: findings from a KOCOSS Cohort. Int J Chron Obstruct Pulmon Dis. (2020) 15:2217–24. 10.2147/COPD.S269579 33061339 PMC7519806

[22] Montserrat-Capdevila J Marsal JR Ortega M Castañ-Abad MT Alsedà M Barbé F. Clinico-epidemiological characteristics of men and women with a new diagnosis of chronic obstructive pulmonary disease: a database (SIDIAP) study. BMC Pulm Med. (2021) 21:44. 10.1186/s12890-021-01392-y 33509131 PMC7842000

[23] Vidaillac C Yong VFL Jaggi TK Soh M-M Chotirmall SH. Gender differences in bronchiectasis: a real issue? Breathe. (2018) 14:108–21. 10.1183/20734735.000218 29875830 PMC5980467

[24] Abid S Xie S Bose M Shaul PW Terada LS Brody SL. 17β-estradiol dysregulates innate immune responses to *Pseudomonas aeruginosa* respiratory infection and is modulated by estrogen receptor antagonism. Infect Immun. (2017) 85:e00422–17. 10.1128/IAI.00422-17 28784925 PMC5607430

[25] Secunda KE Guimbellot JS Jovanovic B Heltshe SL Sagel SD Rowe SM. Females with cystic fibrosis demonstrate a differential response profile to ivacaftor compared with males. Am J Respir Crit Care Med. (2020) 201:996–8. 10.1164/rccm.201909-1845LE 31841644 PMC7159427

[26] Vázquez-Martínez ER García-Gómez E Camacho-Arroyo I González-Pedrajo B. Sexual dimorphism in bacterial infections. Biol Sex Differ. (2018) 9:27. 10.1186/s13293-018-0187-5 29925409 PMC6011518

[27] Bwire GM. Coronavirus: why men are more vulnerable to Covid-19 than women? SN Compr Clin Med. (2020) 4:1–3. 10.1007/s42399-020-00341-w 32838138 PMC7271824

[28] Chan ED Iseman MD. Slender, older women appear to be more susceptible to nontuberculous mycobacterial lung disease. Gend Med. (2010) 7:5–18. 10.1016/j.genm.2010.01.005 20189150

[29] Man WH van Houten MA Mérelle ME Vlieger AM Chu MLJN Jansen N. Bacterial and viral respiratory tract microbiota and host characteristics in children with lower respiratory tract infections: a matched case-control study. Lancet Respir Med. (2019) 7:417–26. 10.1016/S2213-2600(18)30449-1 30885620 PMC7172745

[30] Coburn B Wang PW Diaz Caballero J Clark ST Brahma V Donaldson S. Lung microbiota across age and disease stage in cystic fibrosis. Sci Rep. (2015) 5:10241. 10.1038/srep10241 25974282 PMC4431465

[31] Becklake MR Kauffmann F. Gender differences in airway behaviour over the human life span. Thorax. (1999) 54:1119–38. 10.1136/thx.54.12.1119 10567633 PMC1763756

[32] Barfod KK Vrankx K Mirsepasi-Lauridsen HC Hansen JS Hougaard KS Larsen ST. The Murine lung microbiome changes during lung inflammation and intranasal vancomycin treatment. Open Microbiol J. (2015) 9:167–79. 10.2174/1874285801509010167 26668669 PMC4676059

[33] Roggenbuck M Anderson D Barfod KK Feelisch M Geldenhuys S Sørensen SJ. Vitamin D and allergic airway disease shape the murine lung microbiome in a sex-specific manner. Respir Res. (2016) 17:116. 10.1186/s12931-016-0435-3 27655266 PMC5031331

[34] Pattaroni C Watzenboeck ML Schneidegger S Kieser S Wong NC Bernasconi E. Early-life formation of the microbial and immunological environment of the human airways. Cell Host Microbe. (2018) 24:857–65.e4. 10.1016/j.chom.2018.10.019 30503510

[35] Heirali AA Workentine ML Acosta N Poonja A Storey DG Somayaji R. The effects of inhaled aztreonam on the cystic fibrosis lung microbiome. Microbiome. (2017) 5:51. 10.1186/s40168-017-0265-7 28476135 PMC5420135

[36] Budden KF Gellatly SL Wood DLA Cooper MA Morrison M Hugenholtz P. Emerging pathogenic links between microbiota and the gut–lung axis. Nat Rev Microbiol. (2017) 15:55–63. 10.1038/nrmicro.2016.142 27694885

[37] Marsland BJ Trompette A Gollwitzer ES. The gut–lung axis in respiratory disease. Annals ATS. (2015) 12:S150–6. 10.1513/AnnalsATS.201503-133AW 26595731

[38] Maslowski KM Vieira AT Ng A Kranich J Sierro F Yu D. Regulation of inflammatory responses by gut microbiota and chemoattractant receptor GPR43. Nature. (2009) 461:1282–6. 10.1038/nature08530 19865172 PMC3256734

[39] Koh A De Vadder F Kovatcheva-Datchary P Bäckhed F. From dietary fiber to host physiology: short-chain fatty acids as key bacterial metabolites. Cell. (2016) 165:1332–45. 10.1016/j.cell.2016.05.041 27259147

[40] Dang AT Marsland BJ. Microbes, metabolites, and the gut–lung axis. Mucosal Immunol. (2019) 12:843–50. 10.1038/s41385-019-0160-6 30976087

[41] Chunxi L Haiyue L Yanxia L Jianbing P Jin S. The gut microbiota and respiratory diseases: new evidence. J Immunol Res. (2020) 2020:2340670. 10.1155/2020/2340670 32802893 PMC7415116

[42] Markle JGM Frank DN Mortin-Toth S Robertson CE Feazel LM Rolle-Kampczyk U. Sex differences in the gut microbiome drive hormone-dependent regulation of autoimmunity. Science. (2013) 339:1084–8. 10.1126/science.1233521 23328391

[43] Que LG Stiles JV Sundy JS Foster WM. Pulmonary function, bronchial reactivity, and epithelial permeability are response phenotypes to ozone and develop differentially in healthy humans. J Appl Physiol. (2011) 111:679–87. 10.1152/japplphysiol.00337.2011 21700892 PMC3174797

[44] Sheffield PE Zhou J Shmool JLC Clougherty JE. Ambient ozone exposure and children's acute asthma in New York City: a case-crossover analysis. Environ Health. (2015) 14:25. 10.1186/s12940-015-0010-2 25889205 PMC4373115

[45] Cho Y Abu-Ali G Tashiro H Kasahara DI Brown TA Brand JD. The microbiome regulates pulmonary responses to ozone in mice. Am J Respir Cell Mol Biol. (2018) 59:346–54. 10.1165/rcmb.2017-0404OC 29529379 PMC6189641

[46] Cho Y Abu-Ali G Tashiro H Brown TA Osgood RS Kasahara DI. Sex differences in pulmonary responses to ozone in mice. Role of the microbiome. Am J Respir Cell Mol Biol. (2019) 60:198–208. 10.1165/rcmb.2018-0099OC 30240285 PMC6376411

[47] Kasahara DI Wilkinson JE Cho Y Cardoso AP Huttenhower C Shore SA. The interleukin-33 receptor contributes to pulmonary responses to ozone in male mice: role of the microbiome. Respir Res. (2019) 20:197. 10.1186/s12931-019-1168-x 31455422 PMC6712741

[48] Tashiro H Kasahara DI Osgood RS Brown T Cardoso A Cho Y. Sex differences in the impact of dietary fiber on pulmonary responses to ozone. Am J Respir Cell Mol Biol. (2020) 62:503–12. 10.1165/rcmb.2019-0124OC 31913653 PMC7110971

[49] García-Gómez E González-Pedrajo B Camacho-Arroyo I. Role of sex steroid hormones in bacterial-host interactions. Biomed Res Int. (2013) 2013:928290. 10.1155/2013/928290 23509808 PMC3591248

[50] Chatterjee M van Putten JPM Strijbis K. Defensive properties of mucin glycoproteins during respiratory infections-relevance for SARS-CoV-2. mBio. (2020) 11:e02374–20. 10.1128/mBio.02374-20 33184103 PMC7663010

[51] Williams OW Sharafkhaneh A Kim V Dickey BF Evans CM. Airway mucus. Am J Respir Cell Mol Biol. (2006) 34:527–36. 10.1165/rcmb.2005-0436SF 16415249 PMC2644218

[52] Van Herreweghen F De Paepe K Roume H Kerckhof F-M Van de Wiele T. Mucin degradation niche as a driver of microbiome composition and *Akkermansia muciniphila* abundance in a dynamic gut model is donor independent. FEMS Microbiol Ecol. (2018) 94:1–13. 10.1093/femsec/fiy186 30239657

[53] Lamoureux C Guilloux C-A Beauruelle C Jolivet-Gougeon A Héry-Arnaud G. Anaerobes in cystic fibrosis patients' airways. Crit Rev Microbiol. (2019) 45:103–17. 10.1080/1040841X.2018.1549019 30663924

[54] Ghorbani P Santhakumar P Hu Q Djiadeu P Wolever TMS Palaniyar N. Short-chain fatty acids affect cystic fibrosis airway inflammation and bacterial growth. Eur Respir J. (2015) 46:1033–45. 10.1183/09031936.00143614 26022954

[55] Chotirmall SH Smith SG Gunaratnam C Cosgrove S Dimitrov BD O'Neill SJ. Effect of estrogen on Pseudomonas mucoidy and exacerbations in cystic fibrosis. N Engl J Med. (2012) 366:1978–86. 10.1056/NEJMoa1106126 22607135

[56] Johannesson M Lúdvíksdóttir D Janson C. Lung function changes in relation to menstrual cycle in females with cystic fibrosis. Respir Med. (2000) 94:1043–6. 10.1053/rmed.2000.0891 11127489

[57] Holtrop M Heltshe S Shabanova V Keller A Schumacher L Fernandez L. A prospective study of the effects of sex hormones on lung function and inflammation in women with cystic fibrosis. Ann Am Thorac Soc. (2021). 10.1513/AnnalsATS.202008-1064OC. [Epub ahead of print]. 33544657 PMC12039855

[58] Shaffer L Bozkanat K Lau M Sharma P Sathe M Lopez X. Gender-affirming hormone therapy in cystic fibrosis - a case of new Pseudomonas infection. Respir Med Case Rep. (2021) 21:32:101353. 10.1016/j.rmcr.2021.101353 33537203 PMC7841348

[59] Tyrrell J Harvey BJ. Sexual dimorphism in the microbiology of the CF “Gender Gap”: estrogen modulation of *Pseudomonas aeruginosa* virulence. Steroids. (2020) 156:108575. 10.1016/j.steroids.2019.108575 31901423

[60] Wang Y Cela E Gagnon S Sweezey NB. Estrogen aggravates inflammation in *Pseudomonas aeruginosa* pneumonia in cystic fibrosis mice. Respir Res. (2010) 11:166. 10.1186/1465-9921-11-166 21118573 PMC3006363

[61] Rosenfeld M Davis R FitzSimmons S Pepe M Ramsey B. Gender gap in cystic fibrosis mortality. Am J Epidemiol. (1997) 145:794–803. 10.1093/oxfordjournals.aje.a009172 9143209

[62] Wallis A Butt H Ball M Lewis DP Bruck D. Support for the microgenderome: associations in a human clinical population. Sci Rep. (2016) 6:19171. 10.1038/srep19171 26757840 PMC4725945

